# Vibration Signal Noise-Reduction Method of Slewing Bearings Based on the Hybrid Reinforcement Chameleon Swarm Algorithm, Variate Mode Decomposition, and Wavelet Threshold (HRCSA-VMD-WT) Integrated Model

**DOI:** 10.3390/s24113344

**Published:** 2024-05-23

**Authors:** Zhuang Li, Xingtian Yao, Cheng Zhang, Yongming Qian, Yue Zhang

**Affiliations:** School of Mechanical Engineering, Nantong University, Nantong 226019, China; 13023566697@163.com (Z.L.);

**Keywords:** vibration signal, noise reduction, Chameleon Swarm Algorithm (CSA), Variate Mode Decomposition (VMD), Wavelet Threshold (WT) denoising, slewing bearing

## Abstract

To enhance fault detection in slewing bearing vibration signals, an advanced noise-reduction model, HRCSA-VMD-WT, is designed for effective signal noise elimination. This model innovates by refining the Chameleon Swarm Algorithm (CSA) into a more potent Hybrid Reinforcement CSA (HRCSA), incorporating strategies from Chaotic Reverse Learning (CRL), the Whale Optimization Algorithm’s (WOA) bubble-net hunting, and the greedy strategy with the Cauchy mutation to diversify the initial population, accelerate convergence, and prevent local optimum entrapment. Furthermore, by optimizing Variate Mode Decomposition (VMD) input parameters with HRCSA, Intrinsic Mode Function (IMF) components are extracted and categorized into noisy and pure signals using cosine similarity. Subsequently, the Wavelet Threshold (WT) denoising targets the noisy IMFs before reconstructing the vibration signal from purified IMFs, achieving significant noise reduction. Comparative experiments demonstrate HRCSA’s superiority over Particle Swarm Optimization (PSO), WOA, and Gray Wolf Optimization (GWO) regarding convergence speed and precision. Notably, HRCSA-VMD-WT increases the Signal-to-Noise Ratio (SNR) by a minimum of 74.9% and reduces the Root Mean Square Error (RMSE) by at least 41.2% when compared to both CSA-VMD-WT and Empirical Mode Decomposition with Wavelet Transform (EMD-WT). This study improves fault detection accuracy and efficiency in vibration signals and offers a dependable and effective diagnostic solution for slewing bearing maintenance.

## 1. Introduction

Slewing bearings, which are crucial components in large slewing machinery, significantly influence equipment performance [1]. Their operation in often harsh conditions increases failure risks, with periodic impacts and loads contributing to wear, cracks, and spalling [2]. Consequently, the fault detection and diagnosis of slewing bearings have become a focal point, leveraging statistical measurements to identify concealed fault features in complex signals at the earliest opportunity [3].

Fault diagnosis via vibration signals, which are rich in fault state information, has been a widespread practice over the past decade. The primary methods for diagnosing faults in vibration signals encompass time domain analysis, frequency domain analysis, and time–frequency domain analysis [4]. The complexity of noise in vibration signals obtained from test benches makes noise-reduction techniques crucial for extracting valuable information, positioning these methods as a focal point of current research [5]. Empirical Mode Decomposition (EMD) and Variational Mode Decomposition (VMD) are prominent noise-reduction techniques that decompose signals into multiple modal components [6]. However, EMD often faces challenges such as end effects and mode aliasing during decomposition [7]. In contrast, VMD excels in decomposing non-stationary mixed signals into distinct single-component modes, effectively preventing aliasing by bandwidth control [8]. As an adaptive and entirely non-recursive approach, VMD is extensively applied in denoising non-stationary signals.

The effectiveness of VMD is influenced by two critical input parameters: the modal decomposition number, *K*, and the penalty factor, *α*. Inappropriate settings can result in either over-decomposition or incomplete decomposition of the signal [9], highlighting the importance of choosing these parameters wisely. To identify the optimal combination of VMD input parameters, various optimization algorithms have been employed, including Particle Swarm Optimization (PSO), Gray Wolf Optimization (GWO), Whale Optimization Algorithm (WOA), and Sparrow Search Algorithm (SSA). Xiong et al. [10] combined PSO and VMD to decompose wind speed signals in complex mountainous areas into heterogeneous modal components, considering their complexity and non-stationary nature. Addressing the drawbacks of random weights and thresholds in traditional Elman neural networks, as well as the volatility and non-stationarity of photovoltaic output power signals, Zhang et al. [11] integrated VMD and GWO to enhance the short-term prediction model for photovoltaic output power using Elman neural networks. Wang et al. [12] predicted and judged the health status factors such as tool wear and damage in the early stage, introducing the WOA-VMD-SVM (support vector machine) model with a recognition accuracy higher than the SVM and GA (Genetic Algorithm)–SVM models. Guo et al. [13] developed a hybrid electricity price prediction model based on VMD and SSA for addressing price volatility, demonstrating superior prediction accuracy in the Pennsylvania–New Jersey–Maryland (PJM) electricity market. However, these optimization algorithms share common drawbacks, including susceptibility to local optima, subpar performance in complex, multi-dimensional scenarios, high computational demands, and slow convergence rates. Introduced by Braik in 2021, the Chameleon Swarm Algorithm (CSA) stands out for its excellent convergence speed and accuracy, drawing inspiration from the dynamic hunting behavior of chameleons [14]. Ji and Cao [15] incorporated CSA into mobile robot obstacle avoidance to assist in finding a dependable path for efficient operations. SAID et al. [16] applied CSA to optimize solutions in the economic load scheduling problem and demonstrated through simulations that CSA calculations yielded superior results. But there is limited research on optimizing the two parameters of VMD using CSA to leverage the exceptional convergence speed and accuracy of CSA. Therefore, this study introduces a novel approach by integrating CSA and VMD to analyze the vibration signal of slewing bearings. Nonetheless, the standard CSA is susceptible to local optimization traps, with the quality of its initial population significantly impacting its performance.

To address this, an advanced noise-reduction model, HRCSA-VMD-WT, is designed for effective signal noise elimination. This model innovates by refining the CSA into a more potent Hybrid Reinforcement CSA (HRCSA). Strategies from Chaotic Reverse Learning (CRL), WOA’s bubble-net hunting, and the greedy strategy with the Cauchy mutation are utilized to diversify the initial population, accelerate convergence, and prevent local optimum entrapment, respectively. Furthermore, by optimizing VMD input parameters with HRCSA, Intrinsic Mode Function (IMF) components are extracted and categorized into noisy and pure signals using cosine similarity. Finally, Wavelet Threshold (WT) denoising targets the noisy IMFs before reconstructing the vibration signal from purified IMFs, achieving significant noise reduction. Comparative analysis with other algorithms demonstrates the proposed model’s effectiveness and superiority, showcasing its robust noise-reduction capabilities for vibration signals.

The contributions of this study are summarized as follows:

(a) The study introduces the innovative noise-reduction model, HRCSA-VMD-WT, which addresses the challenge of signal noise in vibration analysis. It significantly improves the Signal-to-Noise Ratio (SNR) and reduces the Root Mean Square Error (RMSE) compared to EMD-WT and CSA-VMD-WT.
(b) The study incorporates the Chaotic Reverse Learning (CRL) strategy, the bubble-net hunting strategy, and the greedy strategy with the Cauchy mutation into standard CSA, enhancing the performance of HRCSA over standard CSA. HRCSA provides a more effective approach for optimizing VMD input parameters.
(c) The study establishes a fatigue test platform integrated with a measurement system utilizing the HRCSA-VMD-WT method for acquiring and processing vibration signals from tested slewing bearings. This offers a practical technical solution for vibration analysis and fault diagnosis in slewing bearings.

The remainder of the paper is organized as follows. Section 2 provides the details of the HRCSA optimization method, including the CSA principle, the CRL strategy, the bubble-net hunting strategy, and the greedy strategy with the Cauchy mutation. The framework of the noise-reduction model, HRCSA-VMD-WT, with its effect evaluation metrics, is presented in Section 3. In Section 4, HRCSA and HRCSA-VMD-WT are both validated based on simulation experiments. The vibration data from a test platform for slewing bearings are used to demonstrate the practical feasibility of the noise-reduction model in Section 5, leading to the conclusions in Section 6.

## 2. Hybrid Reinforcement CSA (HRCSA)

CSA is a new optimization algorithm that can find the optimal solution according to the dynamic behavior of a chameleon hunting prey and has excellent convergence speed and solving accuracy. But at the same time, similar to other heuristic algorithms, the quality of the initial population will affect the performance of the algorithm. When a chameleon in the population is close to the prey, other individuals will approach the chameleon, resulting in the algorithm easily falling into the local optimal solution. In this study, a Hybrid Reinforcement CSA is proposed, which not only guarantees the convergence speed but also improves its ability to jump out of the local optimal solution.

### 2.1. CSA Principle

CSA draws inspiration from the hunting behavior of chameleons. Distinct from the Chameleon Algorithm (CA), which primarily serves as a bottom-up clustering algorithm, CSA is adept at addressing global numerical optimization and constraint problems. Based on the verification using a Markov chain, CSA can reliably converge to the global optimal solution with a probability of 1. The operational mechanism of CSA mimics the chameleon’s prey interaction strategy, which encompasses three main phases: locating prey, tracking prey, and capturing prey [17]. The mathematical model and steps of the algorithm are as follows.

(1) Initial population

Assuming the population size of the chameleons is *n*, the hunt for prey occurs in *m-*dimensional space. The *n × m* matrix *X* corresponding to the chameleons’ positions is defined by Equation (1) [15]:

(1) $$X=\left[\begin{array}{c} x^{1,1}\\ \begin{array}{l} \;\vdots \\ x^{i,1}\\ \;\vdots \\ x^{n,1} \end{array} \end{array}\begin{array}{c} \cdots \\ \begin{array}{l} \ddots \\ \ldots \\ ⋰\\ \cdots \end{array} \end{array}\;\begin{array}{c} x^{1,j}\\ \begin{array}{l} \;\vdots \\ x^{i,j}\\ \;\vdots \\ x^{n,j} \end{array} \end{array}\begin{array}{c} \cdots \\ \begin{array}{l} ⋰\\ \cdots \\ \ddots \\ \cdots \end{array} \end{array}\;\begin{array}{c} x^{1,m}\\ \begin{array}{l} \;\vdots \\ x^{i,m}\\ \;\vdots \\ x^{n,m} \end{array} \end{array}\right]$$

where *x^i,j^* represents the position of the *i*-th chameleon in the *j*-th dimensional space, and 1 ≤ *i* ≤ *n*, 1 ≤ *j* ≤ *m*. We initialize the positions of the chameleon group in *m*-dimensional space using Equation (2):

(2) $$x^{i,j}=lb+r(ub-lb)$$

where *ub* and *lb* represent the upper and lower boundaries of the search space, respectively; *r* denotes a random number; and $r\in \left(0,1\right)$.

(2) Locating prey

The chameleon will constantly change its position during the prey search process, guided by previous location and social experience, and its position-renewal strategy is mathematically described as follows [15].

During the prey search process, the chameleon dynamically adjusts its position, influenced by prior locations and social experience. The strategy for updating its position is mathematically outlined in Equation (3) [14]:

(3) $$x_{t+1}^{i,j}=\left\{\begin{array}{c} x_{t}^{i,j}+p_{1}(P_{t}^{i,j}-G_{t}^{j})r_{2}+p_{2}(G_{t}^{j}-y_{t}^{i,j})r_{1},\;r_{i}\geq P_{p}\\ x_{t}^{i,j}+\mu [(ub-lb)r_{3}+lb]\mathrm{sgn}(rand-0.5),\;r_{i}<P_{p} \end{array}\right.$$

where $x_{t+1}^{i,j}$ is the new position of the *i* chameleon in the *j*-th dimensional space after *t +* 1 iteration; *p*_1_ and *p*_2_ are positive coefficients used to control the ability of algorithm development; $P_{t}^{i,j}$ is the best position of the chameleon *i* in the *j*-th dimensional space in the current *t* iteration; $G_{t}^{j}$ is the global best position in the *j*-th dimensional space in the *t*-th iteration; $r_{i}$ is a random number in the range of (0,1); sgn(*rand* − 0.5) is used to control the direction of rotation of the chameleon, and its value can either +1 or −1; *P_p_* is the probability of the chameleon perceiving things; μ is the search capability parameter, which changes over *t* and is defined by Equation (4):

(4) $$\mu =\gamma e^{{\left(-\frac{\alpha t}{T}\right)}^{\beta }}$$

where *α*, *β* and *γ* are constants; *T* is the maximum number of iterations. According to the research in reference [15], setting *α*, *β* and *γ* to 1, 3.5, and 3, respectively, yields strong search capabilities.

(3) Tracking prey

The chameleon’s ability to rotate its eyes allows it to search for prey across a full 360° range. It updates its position based on the prey’s location, a process mathematically defined in Equation (5) [14]:

(5) $$x_{t+1}^{i}=m(x_{t}^{i}-{\bar{x}}_{t}^{i})+{\bar{x}}_{t}^{i}$$

where $x_{t}^{i}$ is the current position; $\bar{x_{t}^{i}}$ is in the center of the *t* iteration; $x_{t+1}^{i}$ is the new position generated after the rotation matrix is flipped; and *m* is the rotation matrix, denoted by Equations (6) and (7):

(6) $$m=R(\theta ,V_{z1,z2})$$

(7) θ=r·sgn(rand−0.5)π

where *θ* represents the random rotation angle of the chameleon, with *θ* ∈ (−180°,180°) determined by generating a random value *r* within the interval (0,1), and $V_{z1,z2}$ is the vector synthesized from two orthogonal vectors *z*_1_ and *z*_2_ in *m* dimensional space. Chameleons track their prey through eye movements in four stages: (1) Calculate the central position of the current flock. (2) Use a rotation matrix to pinpoint the prey’s position. (3) Form the transformation position through the rotation matrix. (4) Derive the final position by integrating the central position of the current chameleon group.

(4) Capturing prey

In this phase, the chameleons capture their prey via tongue strikes. The chameleon nearest to the prey represents the optimal solution within the current group. The efficiency of prey capture is determined by the velocity of the chameleon’s tongue extension, as detailed in Equation (8):

(8) $$v_{t+1}^{i,j}=v_{t}^{i,j}+c_{1}(G_{t}^{j}-x_{t}^{i,j})r_{1}+c_{2}(P_{t}^{i,j}-x_{t}^{i,j})r_{2}$$

where *c*_1_ and *c*_2_ are cognitive factor constants, and $v_{t}^{i,j}$ is the speed of the *t*-th iteration. Based on the velocity, acceleration, and displacement relationship in rigid body kinematics, Equation (9) governs the chameleon’s updated position at this stage:

(9) $$x_{t+1}^{i,j}=x_{t}^{i,j}+\frac{{\left(v_{t}^{i,j}\right)}^{2}-{\left(v_{t-1}^{i,j}\right)}^{2}}{2a}$$

where *a* is the acceleration, which changes with the number of iterations in a non-linear manner, as described in Equation (10):

(10) $$a=2590\times (1-e^{-\mathrm{lg}t})$$

### 2.2. Chaotic Reverse Learning (CRL) Strategy

Similar to other heuristic optimization algorithms, the quality of the initial population significantly influences the algorithm’s performance, impacting its global search capability and convergence speed. In the standard CSA, population initialization involves using a random number between 0 and 1. However, solely relying on the random function may not ensure the uniform distribution of chameleon individuals in the initial population, potentially causing individuals to be too distant or too close to each other [18]. This scenario increases the risk of the algorithm converging to local optima.

**Figure 1:**
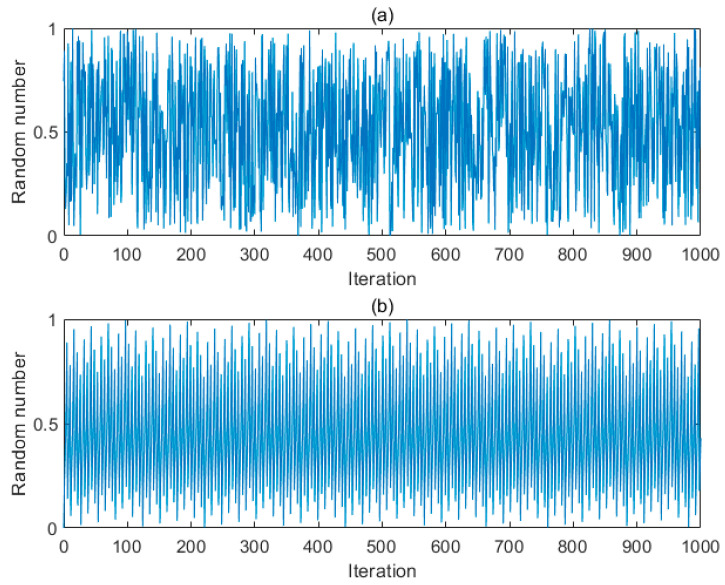
(**a**) Random distribution; (**b**) Circle mapping distribution.

The circle mapping from CRL exhibits strong randomness and ergodicity, enhancing the global search capability of the algorithm through its mapping of population members. The function is defined by Equation (11):

(11) $$y_{i+1}=\mathrm{mod}[y_{i}+0.2-\frac{0.5}{2\pi }\times \sin(2\pi y_{i}),1]$$

where *i* is the sequence number of the chaos variable. Figure 1 illustrates the distribution of the circle mapping and random distribution in a chaotic state. According to Figure 1, circle mapping provides a more uniform randomness, facilitating the generation of varied initial populations.

The expression for calculating the initial population’s position based on circle mapping is shown in Equation (12).

(12) $$x^{i,j}=lb+y_{i}(ub-lb)$$

The reverse learning strategy enhances population diversity and algorithm search capability by generating reverse individuals at each position within the current population. The expression of a new reverse population for the integrated circle mapping is given in Equation (13):

(13) $$X^{i,j}=lb+ub-x^{i,j}$$

where $x^{i,j}$ is the population generated by circle mapping; $X^{i,j}$ is the new population generated by reverse learning. $x^{i,j}$ and $X^{i,j}$ are merged and ranked based on fitness, with the top *n* individuals chosen to form the final population.

### 2.3. Bubble-Net Hunting Strategy

Equation (5) shows that updating chameleons’ positions during prey tracking involves using their central and current positions and adjusting hunting direction with a rotation matrix. While this offers guidance, it introduces randomness and uncertainty. Early iterations may see guidance fail if an individual’s position significantly deviates from the center, impacting the algorithm’s convergence speed. This study incorporates WOA’s bubble-net hunting strategy to enhance global search capabilities, with the position update equation presented in Equation (14):

(14) $$x_{t+1}^{i,j}={\bar{x}}_{t}^{i,j}+D\cdot \cos(2\pi l)\cdot e^{bl}$$

where ${\bar{x}}_{t}^{i,j}$ is the central position of the *t*-th iteration; *b* determines the spiral shape, *b* = 1 here; and *r* and *l* are random numbers in [−1,1]. *D* is the random distance between the central position ${\bar{x}}_{t}^{i,j}$ and the actual position $x_{t}^{i,j}$, which decreases with the number of iterations, denoted as Equation (15):

(15) $$D=\left|2r\cdot {\bar{x}}_{t}^{i,j}-x_{t}^{i,j}\right|$$

where *r* is a random number between 0 and 1. The bubble-net hunting strategy is introduced into the chameleon-tracking prey process. To balance the new algorithm’s global search capabilities and local search efficiency [19], an adaptive dynamic inertial weight *w*(*t*) is introduced. The chameleon’s position update at this stage follows Equation (16):

(16) $$w(t)=\frac{e^{2(1-\frac{t}{T})}-e^{-2(1-\frac{t}{T})}}{e^{2(1-\frac{t}{T})}+e^{-2(1-\frac{t}{T})}}$$

where *T* represents the maximum number of iterations.

The chameleon’s position update at this stage follows Equation (17).

(17) $$x_{t+1}^{i,j}=w(t){\bar{x}}_{t}^{i,j}+D\cdot \cos(2\pi l)\cdot e^{bl}$$

**Figure 2:**
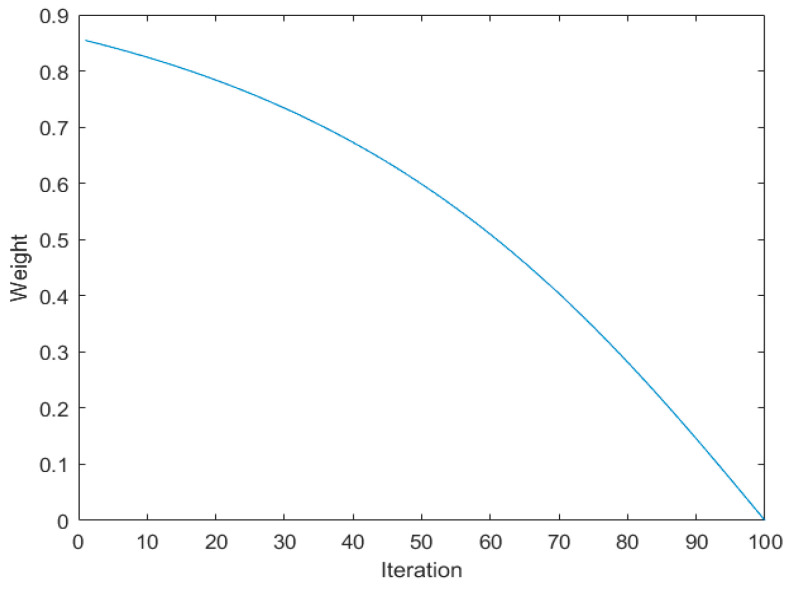
Adaptive dynamic weight curve.

**Figure 3:**
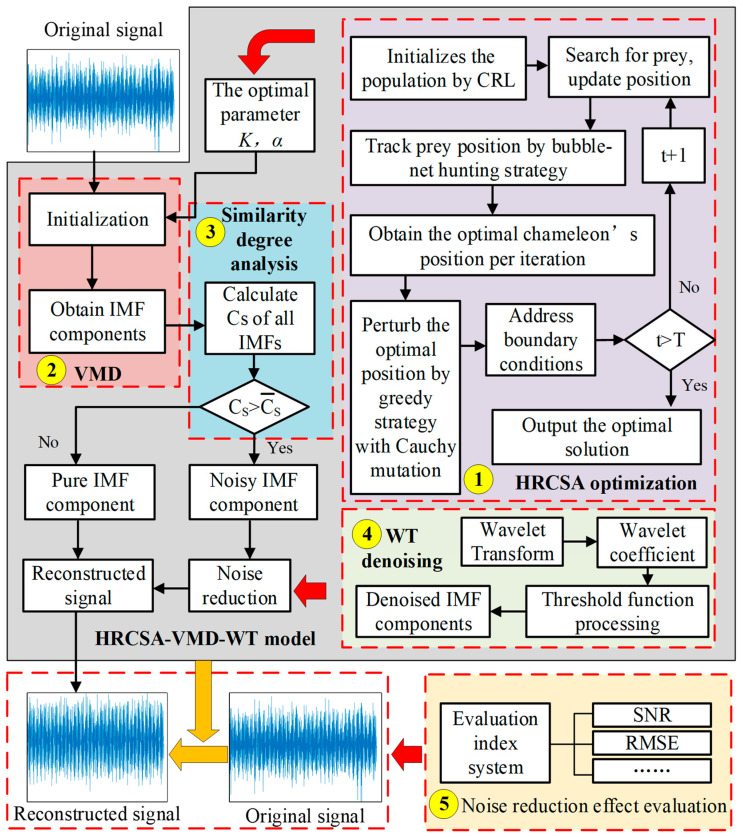
Flow diagram of the HRCSA-VMD-WT noise-reduction method.

Figure 2 shows the change curve of adaptive inertial weight *w*(*t*) with the number of iterations, from which it can be seen that a large inertial weight is used to enhance the global search ability in the early iteration, while a small inertial weight is used to maintain excellent local development ability in the later iteration. Figure 3 illustrates how the adaptive inertial weight varies with the iteration count, using a larger inertial weight early on to boost global search capabilities and a smaller one later to enhance local search efficiency.

### 2.4. Greedy Strategy with Cauchy Mutation

During the algorithm’s later stages, chameleon group members tend to converge near the optimal chameleon’s position, increasing the risk of local optima entrapment. To mitigate this, this study introduces a Cauchy variation strategy that randomly alters the algorithm’s evolutionary direction by perturbing the optimal chameleon’s position per iteration, thereby enhancing the algorithm’s capability to escape local optima. The chameleon’s position post-Cauchy mutation is updated according to Equation (18):

(18) $$x_{t+1}^{new}=x_{t+1}^{best}+cauchy(0,1)\times x_{t+1}^{best}$$

where $x_{t+1}^{best}$ is the optimal chameleon position in the (*t +* 1)-th iteration; *cauchy*(0, 1) is the standard Cauchy distribution; and $x_{t+1}^{new}$ is the chameleon position disturbed by Cauchy variation. Given the uncertainty of the chameleon’s position improvement post-Cauchy variation, a greedy strategy is employed to decide if the optimal chameleon’s position should be updated based on fitness comparisons before and after the disturbance. This strategy is formulated in Equation (19):

(19) $$x_{t+1}^{i,j}=\left\{\begin{array}{cc} x_{t+1}^{new} & \mathrm{if}\;f(x_{t+1}^{new})<f(x_{t+1}^{best})\\ x_{t+1}^{best} & \mathrm{otherwise} \end{array}\right.$$

where *f* is the fitness function or objective function. In this study, the objective function is defined as the minimum envelope entropy, represented by Equations (20) and (21):

(20) $$E_{p}=-\sum_{j=1}^{N}p_{j}\mathrm{lg}p_{j}$$

(21) $$p_{j}=\frac{a(j)}{\sum_{j=1}^{N}a(j)}$$

where *a*(*j*) represents the *j*-th signal data.

## 3. Noise-Reduction Method Framework

### 3.1. Framework of HRCSA-VMD-WT Noise-Reduction Method

The proposed HRCSA-VMD-WT noise-reduction method framework encompasses the noise-reduction model to denoise the vibration signal of slewing bearings and the index system to assess the noise-reduction effect. The noise-reduction model optimizes VMD based on Hybrid Reinforcement CSA and integrates VMD with WT denoising. Figure 3 shows the flow diagram of the HRCSA-VMD-WT noise-reduction method framework. The main parts of the framework are outlined as follows.

(1) HRCSA optimization: HRCSA optimization is used to find the optimal input parameters for VMD. In this study, the standard CSA is adjusted by introducing the CRL strategy, the bubble-net hunting strategy, and the greedy strategy with the Cauchy mutation. The main steps of HRCSA are as follows. (1) Select the minimum envelope entropy as the objective function and use CRL to initialize the population. (2) Update the chameleon position according to bubble-net hunting strategy. (3) Obtain the optimal chameleon position per iteration. (4) Perturb the optimal chameleon’s position per iteration by the Cauchy mutation and update the new chameleon’s position by the greedy strategy that determines whether to update the optimal chameleon’s position. (5) For chameleon individuals beyond the boundary constraint, their position is randomly updated to terminate their tendency to approach the boundary attachment. (6) Obtain the optimal solution of VMD input parameters when the termination condition of the iteration is met.
(2) VMD: VMD is utilized to decompose the original vibration signal of the slewing bearing. Optimal input parameters are input during VMD initialization. The original vibration signal of the slewing bearing is decomposed into *K* IMF components.
(3) Similarity degree analysis: In this study, the cosine similarity degree is employed to categorize each IMF into either a noisy or a pure component. The cosine similarity degree of each IMF component with the original signal is calculated. Based on the average value of the cosine similarity degree of all IMFs, the IMFs with the cosine similarity degree above the average value will be identified as noisy IMFs.
(4) WT denoising: WT denoising is used to eliminate the signal noise of the noisy IMFs. The main steps of WT denoising are as follows. (1) The noisy IMFs are transformed by the wavelet. (2) The wavelet coefficient is calculated. (3) The threshold function handles the wavelet coefficients. (4) The IMF signal with noise is reconstructed after de-noising.

### 3.2. VMD Principle

VMD is a widely used method for processing time-frequency signals, distinguished by its self-adaptive and non-recursive features. It posits that signals can be broken down into linear combinations of eigenmodes with constrained bandwidth [19], effectively turning signal processing challenges into variational model resolution tasks. Within this framework, an input signal, *x*(*t*), is segmented into *K* IMF components based on a predefined decomposition level *K* and a quadratic penalty factor α. In Figure 3, the VMD processes are split into two steps. The first step is “Initialization”, where the range boundary values for the two parameters of the VMD are set, and their optimization combination is determined by HRCSA based on the input *x*(*t*). The second step is “Obtain IMF components”. Throughout this step, the center frequency and bandwidth of each IMF are dynamically adjusted. Equation (22) is the expression of the IMF components [20]:

(22) $$u_{k}(t)=A_{k}(t)\cos[\phi_{k}(t)]$$

where *A_k_*(*t*) is the instantaneous amplitude of IMF component; $\phi_{k}(t)$ is its phase function; *w_k_*(*t*) is its instantaneous phase; and $w_{k}(t)=\phi_{k}(t)$.

In order to obtain the restricted bandwidth of each IMF component, a Hilbert transformation is performed on each IMF component. Subsequently, the frequency of $u_{k}(t)$ is shifted to *w_k_*, resulting in the establishment of a constraint equation, denoted as Equation (23) [21]:

(23) $$\left\{\begin{array}{c} \underset{\left\{u_{k}\right\},\left\{w_{k}\right\}}{\min}\left\{\sum_{k}{\left\|\partial_{t}[(\delta (t)+\frac{J}{\pi t})*u_{k}(t)]e^{-Jw_{k}t}\right\|}_{2}^{2}\right\}\\ s.t.\sum_{k}u_{k}(t)=x(t) \end{array}\right.$$

where $\left\{u_{k}\right\}$ is the set of *K* IMF components, $\{u_{k}\}=\left\{u_{1},\cdots ,u_{k}\right\}$; $\left\{w_{k}\right\}$ is the set of *K* IMF component center frequencies, $\{w_{k}\}=\left\{w_{1},\cdots ,w_{k}\right\}$; δ(t) is the Dirac delta function; the constraint is that the sum of the IMF components equals the input signal *x*(*t*); $\partial_{t}$ is partial derivative operation with respect to *t*; ${\left\|•\right\|}_{2}^{2}$ is the squares of the Euclidean norm; *j* is the imaginary unit, and * is the convolution operation.

To solve $\left\{u_{k}\right\}$ and $\left\{w_{k}\right\}$ above, by introducing Lagrange multipliers λ and penalty factors *α*, the constrained variational problem is transformed into an unconstrained variational problem, and the augmented Lagrange function is obtained by Equation (24) [22]:

(24) $$\begin{array}{l} L(\left\{u_{k}\right\},\left\{w_{k}\right\},\lambda )=\alpha \sum_{k}{\left\|\partial_{t}[(\delta (t)+\frac{J}{\pi t})*u_{k}(t)]e^{-Jw_{k}t}\right\|}_{2}^{2}\\ +{\left\|f(t)+\sum_{k}u_{k}(t)\right\|}_{2}^{2}+\langle \lambda (t),f(t)-\sum_{k}u_{k}(t)\rangle \end{array}$$

where $\langle \lambda (t),f(t)-\sum_{k}u_{k}(t)\rangle$ represents the inner product of λ(t) and $f(t)-\sum_{k}u_{k}(t)$. Alternating Direction Method of Multipliers (ADMM) is used to iteratively optimize $\left\{u_{k}\right\}$, $\left\{w_{k}\right\}$, and λ, to locate the saddle point of the augmented Lagrange function. The iterative process concludes once the specific condition, outlined in Equation (25), is met:

(25) $$\frac{\sum_{k}{\left\|{\hat{u}}_{k}^{n+1}-{\hat{u}}_{k}^{n}\right\|}_{2}^{2}}{\sum_{k}{\left\|{\hat{u}}_{k}^{n}\right\|}_{2}^{2}}<e$$

where ${\hat{u}}_{k}^{n}$ represents the Fourier transform of $u_{k}^{n}$; *e* is the discriminant accuracy; and the value is greater than 0.

### 3.3. Similarity Degree Analysis

Decomposing the original signal with VMD yields *K* Intrinsic Mode Function (IMF) components. This study employs cosine similarity degree to categorize each IMF into either a noisy or a pure component. The calculation of cosine similarity is detailed in Equation (26):

(26) $$Cs(Y_{1},Y_{2})=\left|\frac{\sum_{n=1}^{N}Y_{1}(n)Y_{2}(n)}{\sqrt{\sum_{n=1}^{N}Y_{1}^{2}(n)}\sqrt{\sum_{n=1}^{N}Y_{2}^{2}(n)}}\right|$$

where *Y*_1_ and *Y*_2_ represent the IMF component signal and the original signal, respectively. By mapping these signals into vector space, their similarity is assessed through the cosine of the angle between them in the inner product space. A cosine similarity value close to 1 indicates a high degree of similarity between the components of *Y*_1_ and *Y*_2_, suggesting that the presence of more noisy components in *Y*_1_ necessitates increased noise-reduction efforts.

### 3.4. WT Principle

The Wavelet Threshold (WT) denoising technique involves decomposing the signal into wavelets, eliminating or reducing noise while preserving or enhancing useful signals, and then reconstructing the signal for denoising. Key parameters of the WT method include the wavelet basis function, decomposition layers, threshold value, and the threshold function. This study adopts the widely used Daubechies wavelet basis with five-layer decomposition. Based on the reference [23], a fixed threshold value is employed, detailed in Equation (27):

(27) $$\lambda =\sqrt{2\ln N}$$

where *N* is the data length of the IMF component. In order to eliminate the discontinuity of the threshold function ${\bar{w}}_{j,k}$, and when the absolute value of the wavelet coefficient is greater than λ, ${\bar{w}}_{j,k}$ is able to quickly approach the original value of the wavelet coefficient, ${\bar{w}}_{j,k}$ adopted in this paper is as Equation (28).

(28) $${\bar{w}}_{j,k}=\left\{\begin{array}{c} sign(w_{j,k})\sqrt{{\left|w_{j,k}\right|}^{2}-\lambda^{2}},\;\left|w_{j,k}\right|\geq \lambda \\ 0,\;\left|w_{j,k}\right|<\lambda \end{array}\right.$$

### 3.5. Noise-Reduction Effect Evaluation

This study employs an objective method to evaluate noise-reduction effects, utilizing an index system with specific indicators [24]. Signal-to-Noise Ratio (SNR) and Root Mean Square Error (RMSE) serve as the chosen metrics for assessing the noise-reduction efficacy.

SNR refers to the ratio of the useful signal power and noise signal power, as defined by Equation (29):

(29) $$\mathrm{SNR}=10\mathrm{lg}\frac{\sum_{i=1}^{N}{x_{a}}^{2}(i)}{\sum_{i=1}^{N}{\left[x_{a}(i)-x_{b}(i)\right]}^{2}}$$

where $x_{a}(i)$ is the original signal; $x_{b}(i)$ is the signal after noise reduction.

RMSE is a measure of the difference between predicted values and observed values, as defined by Equation (30):

(30) $$\mathrm{RMSE}=\sqrt{\frac{\sum_{i=1}^{N}{\left[x_{a}(i)-x_{b}(i)\right]}^{2}}{N}}$$

where *N* is the number of observations. 

## 4. Simulation Experiment

To validate the proposed noise-reduction model, HRCSA-VMD-WT, this study introduces simulations and comparative analyses with recent algorithms, including PSO, WOA, and GWO. The validation process is structured into two parts: firstly, assessing HRCSA’s effectiveness, and secondly, evaluating the HRCSA-VMD-WT noise-reduction model. In the simulation experiment, the number of population is 100 and the maximum number of iterations is 100. The main parameters of each algorithm are shown in Table 1.

### 4.1. Simulation Experiment of HRCSA

This study evaluates the algorithms’ performance and reliability using five specific test functions as benchmarks. Functions F1 and F2, characterized as single-peak, assess the algorithms’ convergence speed and precision. Conversely, functions F3 to F5, which are multi-peak, examine their capability to escape local optima. Details and characteristics of these functions are delineated in Table 2.

**Figure 4:**
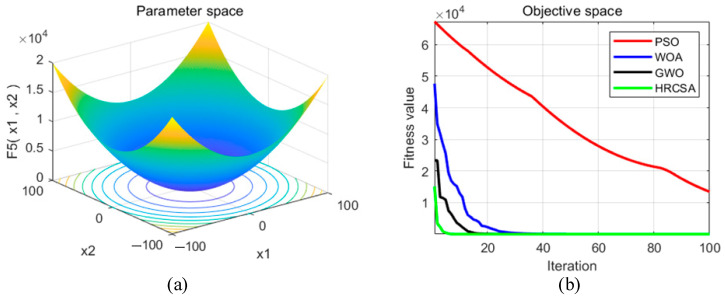
(**a**) Three-dimensional plot of F_1_; (**b**) Convergence curve of different algorithms for F_1_.

**Figure 5:**
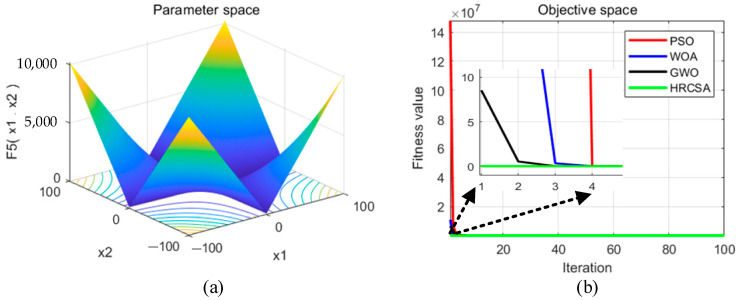
(**a**) Three-dimensional plot of F_2_; (**b**) Convergence curve of different algorithms for F_2_.

**Figure 6:**
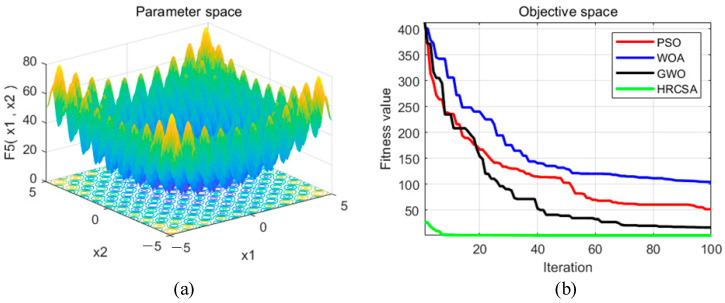
(**a**) Three-dimensional plot of F_3_; (**b**) Convergence curve of different algorithms for F_3_.

**Figure 7:**
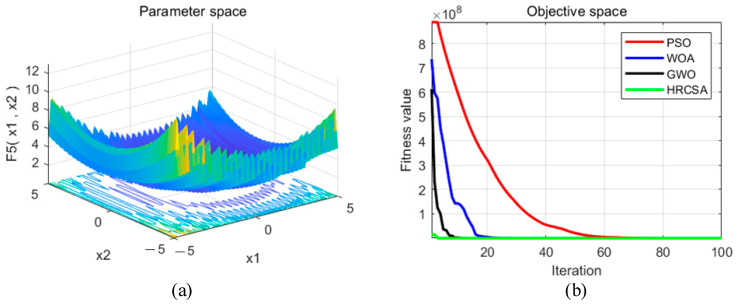
(**a**) Three-dimensional plot of F_4_; (**b**) Convergence curve of different algorithms for F_4_.

**Figure 8:**
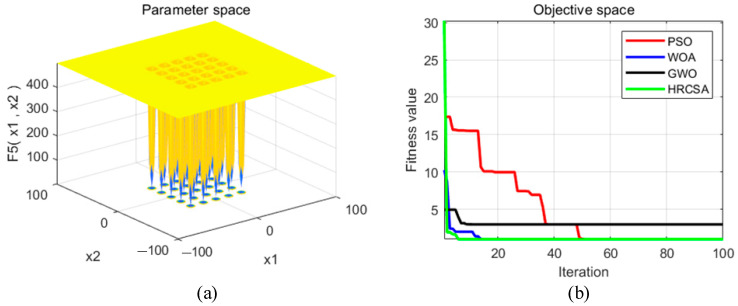
(**a**) Three-dimensional plot of F_5_; (**b**) Convergence curve of different algorithms for F_5_.

Four optimization algorithms were applied to five test functions in thirty independent runs. For each test function, the algorithms determined the optimal solution, average value, and standard deviation. These statistical outcomes are presented in Table 3. Figure 4, Figure 5, Figure 6, Figure 7 and Figure 8 illustrate the analyses: part (a) displays the three-dimensional representation of each test function, while part (b) depicts the iterative performance of each algorithm.

From the three-dimensional graph of the test function, it is evident that F1 and F2 possess only one extreme point, whereas F3 to F5 have multiple extreme points, thereby raising the difficulty of optimization. The results of four optimization algorithms indicate that for the F1 test function, WOA, GWO, and HRCSA successfully identified the optimal solution, whereas the PSO algorithm exhibited a noticeable deviation even after 100 iterations. For the F2 function, all four algorithms reached the optimal solution, yet the PSO’s precision was comparatively lower. Among these, only the HRCSA algorithm could pinpoint the optimal solution for the multimodal F3 function. An analysis of five iterative convergence diagrams reveals that the HRCSA algorithm demonstrates the least initial deviation from the optimal solution, leading to quicker convergence in subsequent iterations. This efficiency is attributed to the integration of a greedy strategy with Cauchy variation, enhancing the accuracy of test results. Consequently, HRCSA exhibits superior stability and precision compared to its counterparts. The PSO algorithm demonstrates superior convergence speed and accuracy in handling low-dimensional problems, albeit with the slowest convergence speed compared to the other three optimization algorithms. In Figure 8, the convergence diagram illustrates that WOA performs similarly to PSO and GWO in terms of convergence speed and accuracy for low-dimensional problems but falls behind GWO for high-dimensional problems. Across Figure 4, Figure 5, Figure 6 and Figure 7, it is evident that GWO outperforms PSO and WOA in terms of convergence speed and accuracy. However, GWO is prone to local optima in low-dimensional problems, as indicated in Figure 8.

### 4.2. Simulation Experiment of HRCSA-VMD-WT

**Figure 9:**
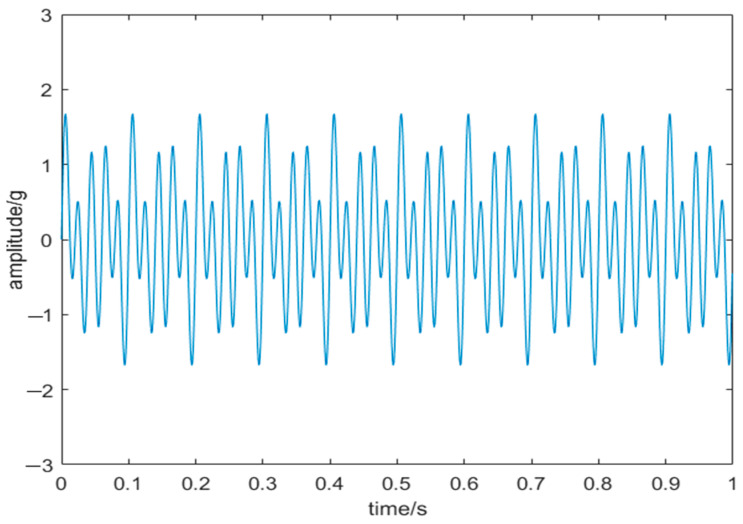
Original signal.

**Figure 10:**
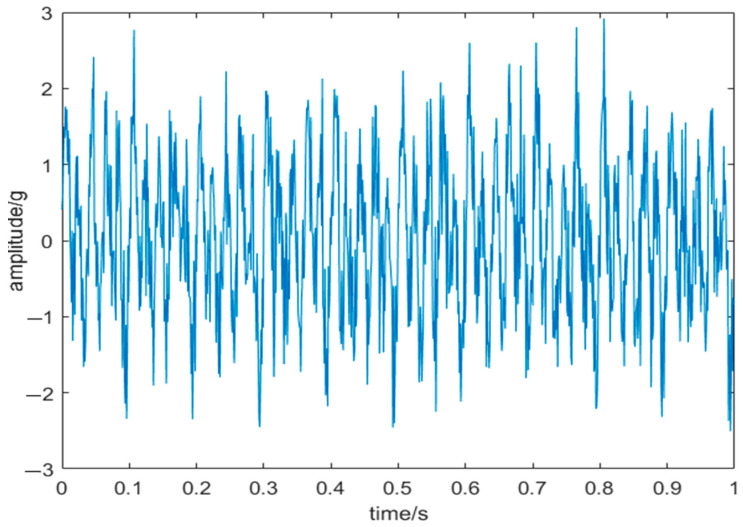
Noisy signal.

To evaluate the proposed noise-reduction model’s performance, simulation signals were analyzed with 1000 data points collected over a duration of 1 s. The original signal *y* and the noisy signal *Y* are defined in Equation (31):

(31) $$\left\{\begin{array}{l} y_{1}=\sin(2\pi f_{1}t)\\ y_{2}=0.5\sin(2\pi f_{2}t)\\ y_{3}=0.4\sin(2\pi f_{3}t)\\ y=y_{1}+y_{2}+y_{3}\\ Y=y+noise \end{array}\right.$$

where *f* is the sampling frequency, with *f*_1_ at 50 Hz, *f*_2_ at 30 Hz, and *f*_3_ at 20 Hz, and *noise* = 0.6*randn*(1, 1000) is white noise generated randomly following a normal distribution. Figure 9 and Figure 10 illustrate the original and corresponding noisy signals, respectively.

**Figure 11:**
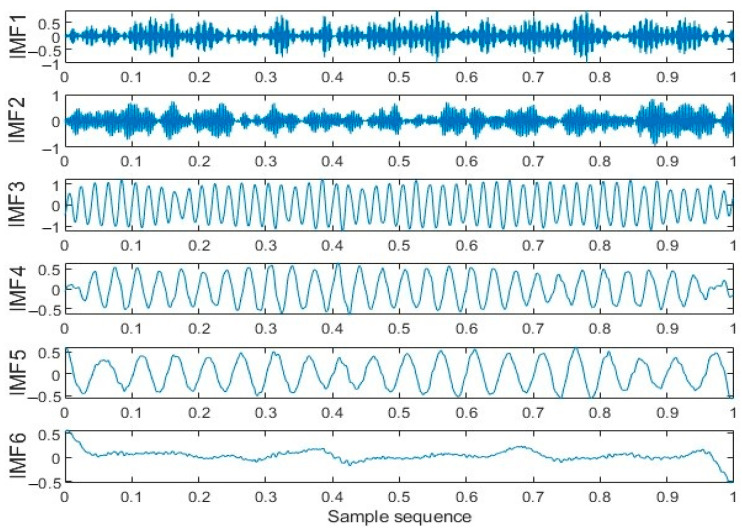
VMD time domain diagram.

**Figure 12:**
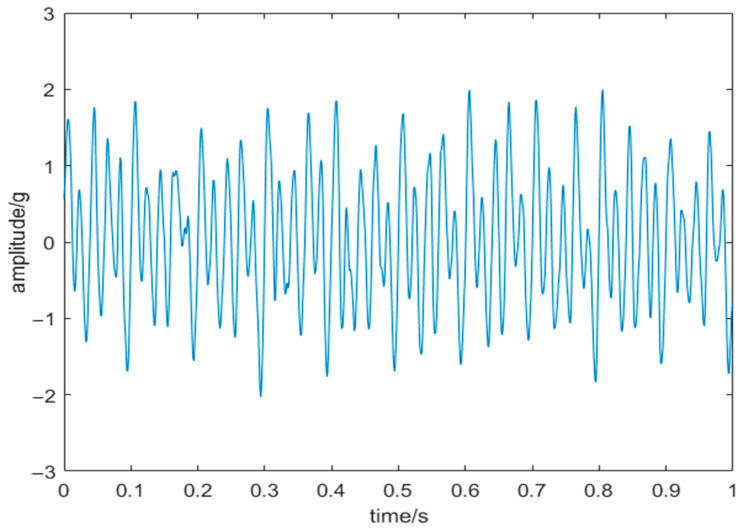
Signal after noise reduction.

The minimum envelope entropy is taken as the objective function of HRCSA. HRCSA was used to derive the optimal VMD parameters, *K* = 6 and *α* = 2495. Figure 11 presents the time-domain representation of the five IMF components post-VMD. Cosine similarity was calculated for each IMF. IMFs with cosine similarity above the average were denoised using WT. The denoised signal is depicted in Figure 12.

This study introduces the EMD-WT and CSA-VMD-WT models alongside HRCSA-VMD-WT to denoise signals. SNR and RSME are used to evaluate the noise-reduction effect of the three models. The corresponding results of these models on SNR and RMSE are shown in Table 4. HRCSA-VMD-WT outperforms EMD-WT, showing a 91.6% improvement in SNR and a 44.7% reduction in RMSE compared to EMD-WT. Furthermore, the analysis highlights the significant influence of the *K* and α parameters on VMD-WT’s noise-reduction capability, underscoring that HRCSA-optimized parameters substantially enhance noise-reduction performance. In the CSA-VMD-WT model, the optimal VMD parameters are *K* = 7 and *α* = 1986, while in the HRCSA-VMD-WT model, the parameters are *K* = 6 and *α* = 2495. Compared with CSA-VMD-WT, HRCSA-VMD-WT improves SNR by 74.9% and reduces RMSE by 41.2%.

## 5. Experimental Verification and Analysis

**Figure 13:**
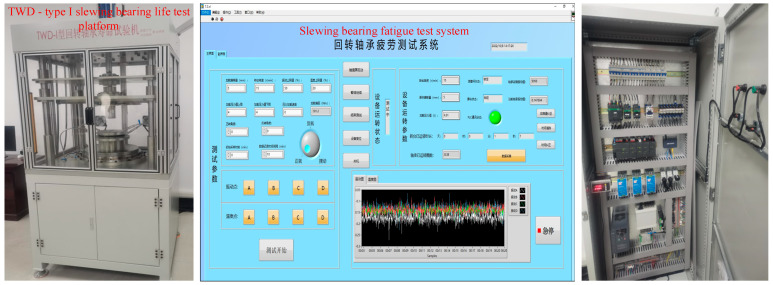
Test platform and its HMI.

A fatigue test platform of slewing bearings is developed to study the dynamic performance of slewing bearings under different operation conditions. The test platform is designed to replicate the axial force and overturning moment encountered by the slewing bearing during operation, inducing relative rotation between the inner and outer rings of the bearing at a specified speed. In the testing process, the inner ring of the bearing is secured to the rotating section of the table using bolts through the connecting flange. This rotating component is powered by a three-phase variable frequency motor via a two-stage synchronous belt reduction system. The upper section of the outer ring of the bearing connects to the upper clamp, where a hydraulic rod applies pressure through the loading head to generate axial force and overturning moment on the bearing. The software component of the test bench is developed using Labview software (Version 20.0), enabling real-time monitoring of the test slewing bearing’s status and recording vibration signals at predefined time intervals. Figure 13 displays the constructed test platform and its Human–Machine Interface (HMI). The initial vibration signals of the tested slewing bearing were obtained using a vibration sensor directly in magnetic contact with the upper clamp on the tested slewing bearing. These initial signals contain various noise components from the mechanical drive system, hydraulic loading system, and other sources. Consequently, when utilizing the captured vibration signals for diagnosing slewing bearing faults, it is essential to conduct noise reduction on the captured vibration signals.

**Figure 14:**
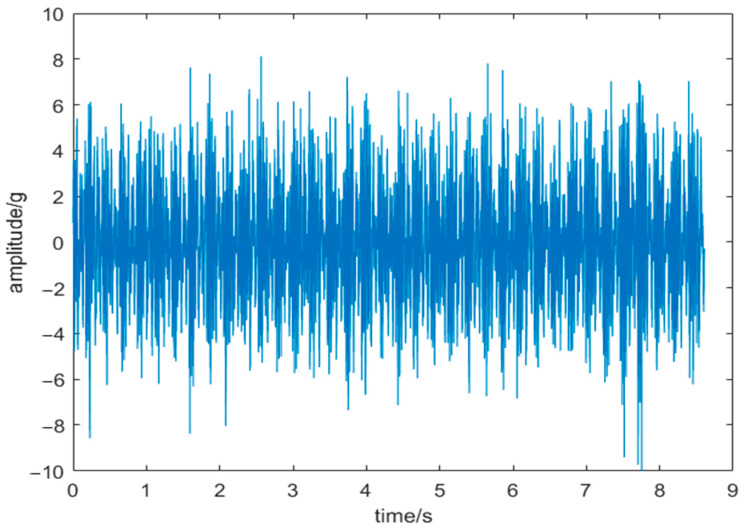
Vibration signal.

**Figure 15:**
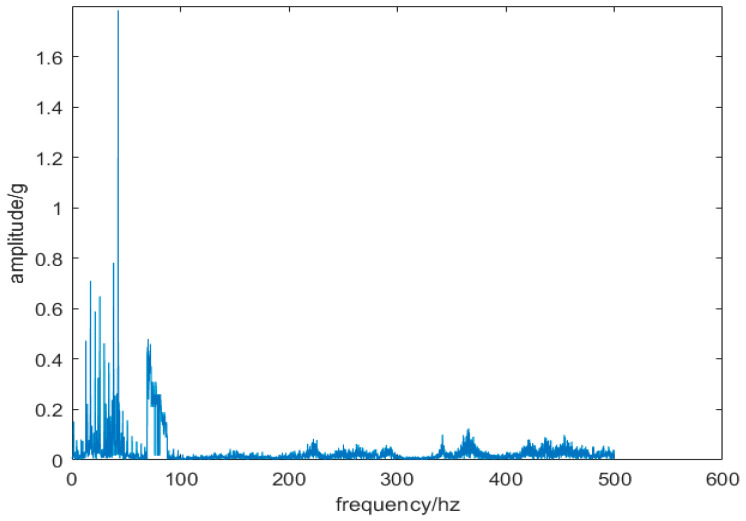
Frequency domain diagram of vibration signal.

This study investigates slewing bearings model 010.25.380 under a 7.6-ton load, with an overturning moment of 1.1402 × 10^7^ N·m and an inner ring rotation speed of 110 r/min. The piezoelectric vibration sensor was selected with a measuring range of ±25 g and a sampling frequency of 1000 Hz. Figure 14 displays the noisy vibration signal of the slewing bearing with the worn inner raceway, while Figure 15 depicts the frequency domain diagram of this signal. In Figure 15, the highest amplitude of the vibration signal is evident at about 42 Hz, with frequencies below 100 Hz being the most prominent. However, accurately determining whether the peak frequency (42 Hz) is a result of noise or bearing failure is challenging due to noise interference in the high-frequency range. This interference may hinder a precise observation of amplitudes at the harmonics of the identified frequency with the highest amplitude.

**Figure 16:**
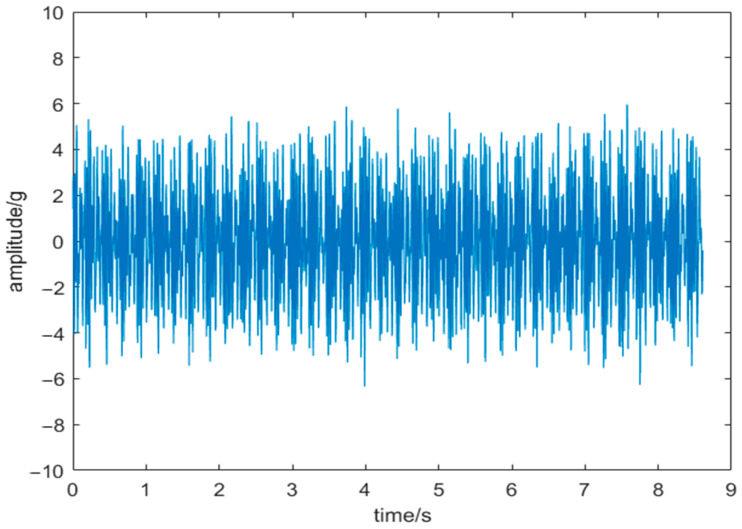
Vibration signal after noise reduction.

**Figure 17:**
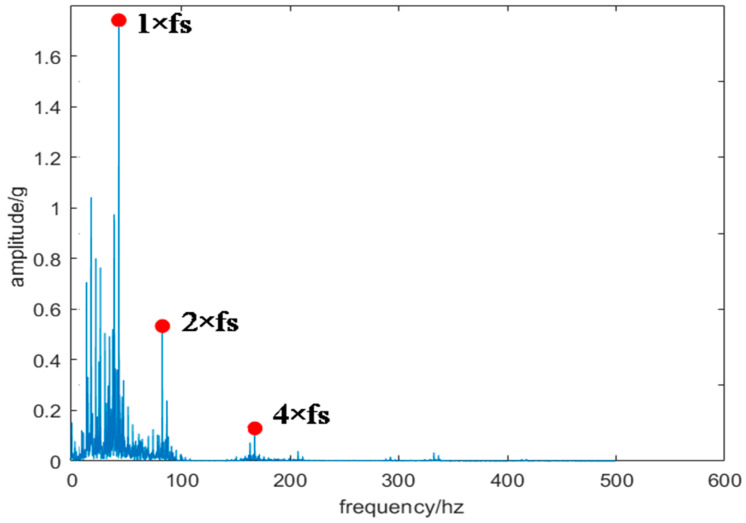
Frequency domain diagram after noise reduction.

Figure 16 depicts the vibration signal curve post-de-noising using the HRCSA-VMD-WT method. Notably, the curve exhibits a reduced amplitude compared to the original signal, highlighting a clearer periodicity in the vibration signal. In Figure 17, the frequency domain diagram of the noise-reduced signal reveals fault frequencies at about 42 Hz, 82 Hz, and 167 Hz that align with the theoretical prediction of the slewing bearing’s inner raceway fault. These findings confirm the practical utility of the HRCSA-VMD-WT method in diagnosing slewing bearing faults.

## 6. Conclusions

This study addresses the issue of excessive random noise in slewing bearing fault vibration signals by introducing the HRCSA-VMD-WT noise-reduction model. The model primarily comprises three parts: HRCSA, VMD, and WT. The novel HRCSA method improves the standard CSA by employing a Chaotic Reverse Learning strategy to enhance the quality of the initial population. It integrates bubble-net hunting and greedy strategies with Cauchy variation to enhance the convergence speed and accuracy of CSA. HRCSA is employed to determine the optimal input parameters *K* and α for VMD. Subsequently, VMD decomposes the noisy vibration signal into a series of IMF components. Noisy IMF components identified using cosine similarity are then denoised through WT, leading to significant noise reduction in the reconstructed signal. Simulation experiments are carried out for both HRCSA and HRCSA-VMD-WT to validate the proposed noise-reduction model. The results show that HRCSA outperforms PSO, WOA, and GWO regarding convergence speed and accuracy, and HRCSA-VMD-WT demonstrates superiority over EMD-WT and CSA-VMD-WT models. Notably, HRCSA-VMD-WT increases the Signal-to-Noise Ratio (SNR) by a minimum of 74.9% and reduces the RMSE by at least 41.2% when compared to both EMD-WT and CSA-VMD-WT. Finally, the initial vibration signals of the tested slewing bearing, obtained from a developed fatigue test bench, are gathered and denoised using the HRCSA-VMD-WT method to demonstrate its application. This study improves fault detection accuracy and efficiency in vibration signals and offers a dependable and effective diagnostic solution for slewing bearing maintenance.

In future research, it will be essential to explore the vibration signal characteristics of slewing bearings under different failure modes. Furthermore, there is a need to further evaluate the potential variations in the noise-reduction effectiveness of the proposed method on vibration signals exhibiting diverse frequency characteristics.

## Figures and Tables

**Table 1:** Main parameter of the algorithms.

Algorithm Name	Main Parameter
PSO	$w_{\max}=0.9$ $,\;w_{\min}=0.2$ $,\;c_{1}=c_{2}=2$
WOA	$a_{\max}=2$ $,\;a_{\min}=0$ , b=1 , p∈[0,1]
GWO	a∈[0,2] $\;\mathrm{and}\;\mathrm{it}\;\mathrm{goes}\;\mathrm{down}\;\mathrm{linearly}\;\mathrm{from}\;2,\;r_{1},r_{2}\in [0,1]$
HRCSA	$c_{1}=2$ $,\;c_{2}=1.8$ $,\;P_{p}=0.1$ , α=1 , β=3.5 , γ=3 $,\;p_{1}=2$ $,\;p_{2}=2$

**Table 2:** Test function feature information.

Functional Formula	Dimensionality	Radius	Optimal Solution
$F_{1}(x)=\sum_{i=1}^{n}x_{i}^{2}$	30	[−100,100]	0
$F_{2}(x)=\sum_{i=1}^{n}\left|x_{i}\right|+\prod_{i=1}^{n}\left|x_{i}\right|$	30	[−10,10]	0
$F_{3}(x)=\sum_{i=1}^{n}\left[x_{i}^{2}-10\cos(2\pi x_{i})+10\right]$	30	[−5.12,5.12]	0
$\begin{array}{l} F_{4}(x)=0.1\{\sin^{2}(3\pi x_{1})+\sum_{i=1}^{n}{\left(x_{i}-1\right)}^{2}[1+\sin^{2}(3\pi x_{i+1})]\\ +{\left(x_{n}-1\right)}^{2}[1+\sin^{2}(2\pi x_{n})]\}+\sum_{i=1}^{n}u(x_{i},5,100,4) \end{array}$	30	[−50,50]	0
$F_{5}(x)={\left[\frac{1}{500}+\sum_{j=1}^{n}\frac{1}{j+\sum_{i=1}^{n}{\left(x_{i}-a_{ij}\right)}^{6}}\right]}^{-1}$	2	[−65,65]	1

**Table 3:** Experimental results of HRCSA and other comparison algorithms on test functions.

Test Function		PSO	WOA	GWO	HRCSA
F1	Optimal solution	1.05 × 10^4^	2.92 × 10^−6^	4.85 × 10^−6^	8.66 × 10^−19^ (best)
	Mean value	1.37 × 10^4^	2.87 × 10^−5^	3.13 × 10^−5^	2.29 × 10^−9^ (best)
	Standard deviation	1.43 × 10^4^	2.78 × 10^−5^	2.91 × 10^−5^	1.24 × 10^−9^
F2	Optimal solution	2.29	1.33 × 10^−4^	4.97 × 10^−4^	3.33 × 10^−6^ (best)
	Mean value	4.62	3.85 × 10^−4^	9.80 × 10^−4^	9.02 × 10^−6^ (best)
	Standard deviation	1.37	2.98 × 10^−4^	3.56 × 10^−4^	1.98 × 10^−6^
F3	Optimal solution	30.18	28.21	8.37	1.38 × 10^−9^ (best)
	Mean value	47.24	77.78	21.48	1.47 (best)
	Standard deviation	8.20	33.36	9.65	1.69
F4	Optimal solution	2.85	0.43	0.31	1.12 × 10^−10^ (best)
	Mean value	6.29	1.09	0.83	9.37 × 10^−3^ (best)
	Standard deviation	1.94	0.37	0.31	0.02
F5	Optimal solution	0.99 (best)	0.99 (best)	0.99 (best)	0.99 (best)
	Mean value	0.99 (best)	1.13	2.83	0.99 (best)
	Standard deviation	1.64 × 10^−10^	0.50	2.71	3.94 × 10^−11^

**Table 4:** Noise-reduction performance table of three models.

Metrics	EMD-WT	CSA-VMD-WT	HRCSA-VMD-WT
SNR	5.5866	6.12	10.703
RMSE	0.4413	0.415	0.244

## Data Availability

The data presented in this study are available upon request from the authors.
